# Integrating tuberculosis and antimicrobial resistance control programmes

**DOI:** 10.2471/BLT.17.198614

**Published:** 2018-02-05

**Authors:** Rumina Hasan, Sadia Shakoor, Johanna Hanefeld, Mishal Khan

**Affiliations:** a Department of Pathology & Laboratory Medicine, Aga Khan University, Stadium Road, PO Box 3500, Karachi 74800, Pakistan.; b Department of Global Health & Development, London School of Hygiene and Tropical Medicine, London, England.

## Abstract

Many low- and middle-income countries facing high levels of antimicrobial resistance, and the associated morbidity from ineffective treatment, also have a high burden of tuberculosis. Over recent decades many countries have developed effective laboratory and information systems for tuberculosis control. In this paper we describe how existing tuberculosis laboratory systems can be expanded to accommodate antimicrobial resistance functions. We show how such expansion in services may benefit tuberculosis case-finding and laboratory capacity through integration of laboratory services. We further summarize the synergies between high-level strategies on tuberculosis and antimicrobial resistance control. These provide a potential platform for the integration of programmes and illustrate how integration at the health-service delivery level for diagnostic services could occur in practice in a low- and middle-income setting. Many potential mutual benefits of integration exist, in terms of accelerated scale-up of diagnostic testing towards rational use of antimicrobial drugs as well as optimal use of resources and sharing of experience. Integration of vertical disease programmes with separate funding streams is not without challenges, however, and we also discuss barriers to integration and identify opportunities and incentives to overcome these.

## Résumé

De nombreux pays à revenu faible et intermédiaire qui sont confrontés à une forte résistance aux antimicrobiens ainsi qu’à la morbidité associée, due à l'inefficacité des traitements, sont aussi fortement touchés par la tuberculose. Ces dernières décennies, de nombreux pays ont mis au point des systèmes efficaces d'information et de laboratoire afin de combattre la tuberculose. Dans cet article, nous décrivons la manière dont les systèmes existants des laboratoires spécialisés dans la tuberculose peuvent être élargis afin d'intégrer des fonctions applicables à la résistance aux antimicrobiens. Nous montrons comment cet élargissement des services pourrait contribuer au dépistage de la tuberculose et aux capacités des laboratoires par l'intégration de services de laboratoire. Nous faisons par ailleurs le point sur les synergies entre les stratégies de haut niveau sur la tuberculose et la lutte contre la résistance aux antimicrobiens. Celles-ci offrent des possibilités pour l'intégration de programmes et illustrent la manière dont l'intégration au niveau de la prestation des services de diagnostic pourrait se faire en pratique dans les régions à revenu faible et intermédiaire. L'intégration pourrait apporter de nombreux bénéfices mutuels, comme l'expansion plus rapide des tests de diagnostic en vue d'une utilisation rationnelle des médicaments antimicrobiens, d'une utilisation optimale des ressources et d'un partage d'expérience. L'intégration de programmes verticaux de lutte contre les maladies, qui ont des sources de financement différentes, n'est cependant pas chose simple. Nous évoquons également les obstacles à cette intégration ainsi que les perspectives et les mesures incitatives pour les surmonter.

## Resumen

Muchos países de ingresos bajos y medianos que enfrentan altos niveles de resistencia a los antimicrobianos, así como la morbilidad asociada por un tratamiento ineficaz, también presentan una alta incidencia de tuberculosis. En las últimas décadas, muchos países han desarrollado sistemas efectivos de laboratorio e información para el control de la tuberculosis. En este documento describimos cómo los sistemas de laboratorio de tuberculosis existentes pueden ampliarse para dar cabida a las funciones de resistencia a los antimicrobianos. Mostramos cómo dicha expansión en los servicios puede beneficiar la búsqueda de casos de tuberculosis y la capacidad de laboratorio a través de la integración de los servicios de laboratorio. Resumimos las sinergias entre las estrategias de alto nivel sobre la tuberculosis y el control de la resistencia a los antimicrobianos. Estos proporcionan una plataforma potencial para la integración de programas e ilustran cómo la integración en el nivel de prestación de servicios de salud para los servicios de diagnóstico podría ocurrir en la práctica en un entorno de ingresos bajos y medianos. Existen muchos beneficios mutuos potenciales de la integración, en términos de una mejora acelerada de las pruebas de diagnóstico hacia el uso racional de los medicamentos antimicrobianos, así como el uso óptimo de los recursos y el intercambio de experiencias. Sin embargo, la integración de programas de enfermedades verticales con flujos de financiación separados no está exenta de desafíos, y también examinamos los obstáculos a la integración e identificamos oportunidades e incentivos para superarlas.

## ملخص

تعاني العديد من البلدان منخفضة ومتوسطة الدخل – والتي تواجه مستويات مرتفعة من مقاومة مضادات الميكروبات، والأمراض المصاحبة الناتجة عن العلاج غير الفعال – من معدلات مرتفعة للإصابة بمرض السل. وعلى مدى العقود الأخيرة قامت العديد من البلدان بتطوير أنظمة مختبرات ومعلومات فعالة لمكافحة مرض السل. ونصف في هذا التقرير كيفية توسيع أنظمة المختبرات الموجودة لتستوعب وظائف مقاومة مضادات الميكروبات. ونوضح كيف أن هذا التوسع في الخدمات قد يفيد في الكشف عن حالات الإصابة بمرض السل وفي القدرات المختبرية من خلال دمج خدمات المختبرات. وقد قمنا كذلك بتلخيص أوجه التآزر بين الاستراتيجيات رفيعة المستوى المتبعة مع مرض السل وبين مكافحة مضادات الميكروبات. وقد ساهم ذلك في تقديم منصة محتملة لدمج البرامج وتوضيح إمكانية التطبيق العملي للدمج على مستوى تقديم الخدمات الصحية للخدمات التشخيصية في بيئة ذات دخل منخفض ومتوسط. وتوجد العديد من المنافع المتبادلة المحتملة للدمج وذلك من حيث تسريع وتيرة التوسع في نطاق الاختبارات التشخيصية نحو الاستخدام الرشيد للعقاقير المضادة للميكروبات فضلًا عن الاستخدام الأمثل للموارد وتبادل الخبرات، إلا أن دمج البرامج الرأسية لمكافحة المرض مع قنوات التمويل المنفصلة ليس بالأمر الذي يخلو من التحديات، وقد قمنا كذلك بمناقشة عوائق الدمج وتمييز الفرص والحوافر للتغلب على الأمر.

## 摘要

许多中低收入国家面临着高水平的抗菌素耐药性，由于治疗效果不佳而造成的相关并发症，以及较高的结核病负担。近几十年来，许多国家建立了有效的结核病控制实验室和信息系统。本文中，我们描述如何扩大现有的结核病实验室系统，以适应抗菌素的耐药功能。我们展示了如何通过实验室服务的整合来扩大服务范围，从而有利于结核病病例发现与实验室检测能力。我们进一步总结了结核病与抗菌素耐药性控制高层战略之间的协同作用。这些为方案的整合提供一个潜在的平台，并且说明如何在低收入和中等收入地区通过实践在医疗诊断服务提供层面进行整合。一体化存在着许多潜在的互惠利益，包括加速推广合理使用抗菌药物的诊断检测，以及优化利用资源和分享经验。纵向疾病方案与单独资金流的整合并非没有挑战，然而，我们还讨论了整合所面临的障碍，并找出克服这些障碍的机会和激励措施。

## Резюме

Многие страны с низким и средним уровнем дохода, столкнувшиеся с высоким уровнем устойчивости к противомикробным препаратам и связанной с ней распространенностью случаев неэффективности лечения, помимо этого, имеют высокую заболеваемость туберкулезом. За последние десятилетия во многих странах были разработаны эффективные лабораторные и информационные системы борьбы с туберкулезом. В этой статье мы описываем, как существующие лабораторные системы для диагностики туберкулеза могут быть расширены путем включения методов определения лекарственной устойчивости к противомикробным препаратам. Мы демонстрируем, как такое расширение сферы обслуживания может способствовать выявлению случаев туберкулеза и укреплению лабораторного потенциала за счет интеграции лабораторных услуг. Далее мы подводим итоги синергии между стратегиями высокого уровня по борьбе с туберкулезом и устойчивостью к противомикробным препаратам. Они обеспечивают потенциальную платформу для интеграции программ и иллюстрируют, каким образом на практике в условиях низкого и среднего уровня дохода можно было бы обеспечить интеграцию на уровне предоставления медицинских услуг для диагностических служб. Существует много аспектов потенциальной взаимной выгоды от интеграции с точки зрения ускоренного расширения диагностического тестирования в направлении рационального использования противомикробных препаратов, а также оптимального использования ресурсов и обмена опытом. Однако интеграция вертикальных программ борьбы с заболеваниями с раздельными потоками финансирования не лишена проблем, поэтому мы также обсуждаем препятствия на пути интеграции и выявляем возможности и стимулы для их преодоления.

## Introduction

Public health programmes that address the threats of antimicrobial resistance and of tuberculosis are major contributors towards gains in global health.^1^^,^^2^ Unlike tuberculosis, antimicrobial resistance is not specifically mentioned in the health targets of United Nations’ sustainable development goal 3.^3^ Both health issues, however, are encompassed in the overarching goal of ensuring healthy lives and promoting well-being for all. The globally endorsed End Tuberculosis strategy^4^ and Global Action Plan on Antimicrobial Resistance^5^ also agree on universal health coverage and collaboration between diverse stakeholders to achieve their objectives.

Despite potential synergies between them, antimicrobial resistance and tuberculosis have until recently been positioned as separate global health issues, and efforts aimed at controlling both remain primarily vertical (disease-specific). Integration across programme components has therefore been limited, possibly leading to economic inefficiencies and suboptimal service delivery. This is exemplified most clearly by the initial decision to exclude Mycobacterium* tuberculosis* from the global priority list of antibiotic-resistant bacteria,^6^ even though estimates suggest that by the year 2050 drug resistant tuberculosis will be responsible for 2.6 million of the total 10 million annual deaths associated with antimicrobial resistance.^1^^,^^7^ The protests and concerns raised following this decision eventually led to the inclusion of M.* tuberculosis* within the priority list, highlighting the importance of integrating activities aimed at addressing both health issues.^8^ A non-systematic review of studies on integration of programmes on maternal and child health, human immunodeficiency virus (HIV), sexually transmitted infections and tuberculosis found that integration increased uptake of services.^9^

Several antimicrobial agents used to treat tuberculosis are also used for management of other infectious diseases. These include fluoroquinolone antibiotics, which are used not only for tuberculosis, but also for respiratory, urinary and enteric infections. A systematic review and meta-analysis found that use of fluoroquinolones in patients with respiratory infections delayed the diagnosis of tuberculosis by nearly 2 weeks,^10^ thus emphasizing the interdependence of antimicrobial resistance and tuberculosis control efforts. Given such overlap, exposure to antimicrobial drugs risks development of resistance in other microorganisms.^11^

In this paper we summarize some opportunities and challenges to integration of tuberculosis and antimicrobial resistance programmes. We first summarize the synergies between high-level strategies on tuberculosis and antimicrobial resistance control. These provide a potential platform for the integration of programmes and illustrate how integration at the health-service delivery level for diagnostic services could occur in practice in a low and middle-income setting. We then discuss barriers to integration and identify opportunities and incentives to overcome these.

## Synergies between programmes

Both the End Tuberculosis strategy and Global Action Plan on Antimicrobial Resistance aim to improve health and control infectious diseases and, in particular, to limit the spread of drug resistance. Therefore, despite differences in their organizational structure and funding streams, integrating certain activities will result in better use of resources and increase the likelihood of achieving mutual goals. The End Tuberculosis strategy already recognizes the importance of collaboration with other initiatives and programmes;^4^ in most countries, for example, tuberculosis programmes have experience of collaboration with HIV programmes. Similarly, many antimicrobial resistance programmes are being built on a One Health approach,^12^ recognizing the importance of engaging multiple partners, including those outside of the human health sector.

Partial integration between individual health programmes can be achieved through linkages and collaborations, but full integration requires integration across the components of governance, financing, service delivery and information systems.^13^ Integration between tuberculosis control and antimicrobial resistance programmes at the global level could promote shared activities within countries to achieve mutual benefits for both programmes (Box 1).

Box 1 **Benefits of integration between programmes for tuberculosis control and antimicrobial resistance**

- Efficient use of resources currently allocated to separate tuberculosis control and antimicrobial resistance programmes towards coordinated prevention and control strategies.
- Sharing of expertise, local experience and existing resources, such as staff and health facilities, to enhance outcomes for both tuberculosis control and antimicrobial resistance programmes.
- Development of synergistic technical packages covering clinical guidelines, diagnostic pathways and tools, infection control and prevention, and evidence-based priority interventions. These could work towards controlling resistance in the *Mycobacterium tuberculosis* complex as well as bacteria in the global list of antibiotic-resistant priority pathogens.^6^
- Greater advocacy and political attention for both tuberculosis and antimicrobial resistance. Collaboration could intensify efforts towards improving the quality of care delivered by informal health-care providers, regulating the pharmaceutical industry and controlling the use of growth promoters in the veterinary industry.
- Reduced reliance on external resources, through integrating tuberculosis control and antimicrobial resistance programmes within the national structures of high-burden countries. In this way common goals would be safeguarded through a strengthened oversight mechanism.

## Integration of laboratory services

Over the last decade those involved in tuberculosis control have developed important new diagnostic tools and established quality-assured laboratory systems. As a result, detection of tuberculosis and in particular drug-resistant tuberculosis has greatly increased.^2^ Tuberculosis control planners have experience in developing laboratory systems towards better quality assurance systems (such as in handling of sputum smears), standardized record-keeping and logistics support (including internet connectivity, reporting to national programmes, supply chain management and coordination of laboratory functions). These experiences could be leveraged to strengthen diagnostic laboratories involved in antimicrobial resistance testing and surveillance. Indeed, the major initial focus of antimicrobial resistance control strategies is on surveillance, with large investments now being made to strengthen the often weak surveillance of antimicrobial resistance in low- and middle-income countries.^14^

A tuberculosis laboratory network generally has a tiered structure,^15^ with microscopy at the basic level, mycobacterial culture at the intermediate level, and culture as well as drug sensitivity testing at the reference laboratory level. Peripheral laboratories can offer point-of-care tests that will help to decrease antibiotic misuse by establishing when diseases have a viral cause. At the intermediate level, laboratories can share culture and antibiotic susceptibility testing capacities to provide public health facilities with appropriate antimicrobial resistance diagnostics. Reference laboratories can contribute to confirmation of bacterial resistance and to surveillance for emerging resistance mechanisms in pathogens. Laboratories in the tuberculosis network have well-established quality management systems and biomedical and biosafety infrastructures and, by using the same facilities for antimicrobial resistance, can bypass the need to create expensive systems in new laboratories dedicated to antimicrobial resistance.

**Fig. 1:**
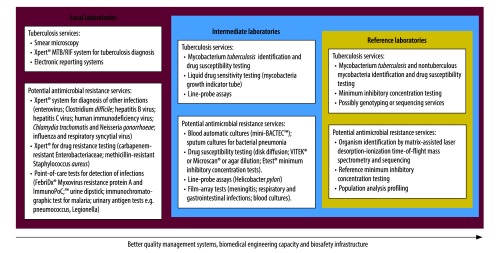
Diagnostic tools currently in use at different levels of tuberculosis diagnostic facilities

Notes: The following are trademark technologies: Xpert® (Cepheid, Sunnyvale, United States of America; USA); FebriDx® (RPS Diagnostics; Sarasota, USA); ImmunoPoC (MeMed Diagnostics, Israel); BACTEC (Becton Dickinson Diagnostics, Sparks, USA); VITEK® (bioMèrieux, Marcy-l'Étoile, France); Microscan® (Beckman Coulter, Brea, USA); Etest® (bioMérieux, Marcy l’Etoile, France).

Such a structure lends itself well to close cooperation and integration with antimicrobial resistance programmes. Several diagnostic tools currently in use for diagnosis of antimicrobial resistance, as well as existing infrastructure and human resources, could be adapted to facilitate the integration of services, and delivered in accordance with the level and expertise available at the relevant tuberculosis laboratories (Fig. 1). In remote areas where laboratory access for diagnosis of infectious diseases is limited, services provided by the most basic tuberculosis microscopy centres could be expanded to include point-of-care testing for common infections. Some examples of point-of-care tests that could be incorporated into existing tuberculosis diagnostic services include malaria diagnosis, microscopy or dipstick testing for urinary tract infections as well as pneumococcal and Legionella antigen tests. This approach would be strengthened when combined with referral of specimens for culture and sensitivity testing and initiation of appropriate treatment to control further spread of resistant organisms. Recently, rapid molecular tests for tuberculosis are being added, particularly at the intermediate and reference levels, but in some cases also at the basic laboratory level. This system is underpinned by logistics support and greater efforts to expand connectivity for reporting and monitoring within the network. At the international level, the tuberculosis laboratory network is supported by several supranational reference laboratories that provide training and on-the-ground assistance and advice as required.

Increasing the breadth of services provided by tuberculosis laboratories could be used to strengthen antimicrobial resistance diagnostic testing and surveillance. Currently, one of the most widely used rapid molecular test for detection of M.* tuberculosis* is Xpert® MTB-RIF (Cepheid, Sunnyvale, United States of America). The Xpert® technology, however, could also be used for the rapid diagnosis of several other bacterial and viral infections. The repertoire of infectious disease diagnostics is constantly expanding, based on new technologies including microfluidics^16^ and film arrays.^17^^,^^18^ These could easily be placed in integrated intermediate level laboratories, with a wider test menu towards guided antimicrobial therapy. Finally, many of the supranational tuberculosis reference laboratories have already confirmed that they could expand susceptibility testing for other pathogens if funding were available.^19^ Their expanded role could be leveraged as an opportunity towards self-sustainability by adding to the core competency of each laboratory and also an expanded role for tuberculosis laboratory networks.

Another advantage is that the broader infrastructure could be shared between tuberculosis and antimicrobial resistance programmes. Low-resource settings face infrastructure challenges to providing laboratory services. These include shortages of trained laboratory staff; lack of access to biomedical technical support;^20^ problems with installing, validating, certifying and servicing laboratory equipment; difficulties in specimen transport;^21^ difficulties in data connectivity and management; and challenges to maintaining biosafety levels.^22^^,^^23^ While laboratory networks can be resource-intensive and expensive to run, they do lend themselves to serving more than one health programme, allowing for optimal use of resources. For example, establishment of an efficient and far-reaching specimen referral network has been explored by investigators in Ethiopia and Uganda, and shown to be effective for multiple diseases, including tuberculosis, HIV and hepatitis.^21^^,^^24^ Therefore, establishing shared laboratory spaces, equipment and supplies, human resources and transport systems would be mutually beneficial to both tuberculosis and antimicrobial resistance programmes and improve universal access to diagnostics for the population served.

## Benefits of integrated services

Integration of certain services in joint laboratories could have benefits for both tuberculosis and antimicrobial resistance programmes. In low-resource settings, expanding the scope of tests within the existing tuberculosis laboratory network would increase patients’ access to diagnostics and encourage rational use of antimicrobials. A recent study on the impact of rapid diagnostic tests for malaria in Africa and Asia demonstrated that while rapid diagnosis reduced antimalarial drug use, it also resulted in over-prescription of antimicrobial agents.^25^ This highlights the importance of not only enhancing access to diagnostics, but also coordinating between disease-specific laboratory networks and antimicrobial resistance control programmes.

Integration will also enhance the capacity of the tuberculosis laboratory network, enabling the facilities and staff to function beyond a single disease area, and thereby serve a larger population of patients. Broadening the patient population served by joint laboratories for tuberculosis and antimicrobial resistance may also help to address the challenge of low research and development funding for tuberculosis diagnostics.^26^ As tuberculosis progresses towards elimination, for-profit companies see limited scope for financial returns on developing new diagnostics for the disease. Investing in diagnostics may be more attractive if companies are able to cater to a larger population with emerging diseases of various etiologies, For example, industry reports estimate that the market value of diagnostics for infectious diseases was worth 14.45 billion United States dollars (US$) in 2016 and expected to reach US$ 21.13 billion by 2021, with the global worth of point-of-care diagnostics expected to reach US$ 1.9 billion by 2025.^27^^,^^28^ With sufficient investment in research and development, there are opportunities for advancements in laboratory medicine. 

Many of the new tests being developed (including those for tuberculosis) are of low complexity and performed near the patient or at the point of care,^29^ which is more convenient and less costly for patients. The focus on patient-centred approaches has also led to the development of multiplex devices designed to rapidly detect a variety of bacterial, viral or fungal pathogens in a single test.^30^ Currently many of these technologies are of moderate complexity, requiring technical expertise that make them more suitable for intermediate or referral laboratories. Developments are underway, however, to make such tests more affordable and to bring them nearer to the point of care.

Newer, more expensive antibiotics are being developed to replace those made redundant due to high levels of drug resistance. From the perspective of an antimicrobial resistance programme these developments will also increase the need to improve access to effective diagnostic tools to rule out differential diagnoses.

## Barriers to integration

A longstanding challenge is how to integrate individual vertical disease control programmes with other vertical programmes and into primary health-care services.^31^^,^^32^ Concerns about the effects of integration on disease-specific funding and on human resources are common across many vertical programmes, such as those for tuberculosis, malaria and HIV.^33^ In the case of tuberculosis and antimicrobial resistance, challenges to integration may arise because powerful stakeholders (such as the Global Fund to Fight AIDS, Tuberculosis and Malaria for tuberculosis and the Global Health Security Agenda focused on antibiotic resistance) largely operate independently of each other. Increasing integration between programmes would, by definition, require some relinquishing of disease-specific resources to a common fund. The efficiencies achieved from joint service delivery would also likely result in job losses if human resource posts are merged, for example among laboratory staff who can perform diagnosis for both antimicrobial resistance and tuberculosis, and this could be a source of conflict.

With different funding and accountability systems, the specific targets and institutional structures of programmes at the country level are also likely to be different. Coordination and communication across national tuberculosis control programmes, surveillance agencies and laboratory services departments will be essential. This will require governance structures at the global and national levels so as to better integrate activities between programmes. Vertical programmes often work towards very focused targets.^34^ Therefore, ensuring shared responsibility for mutually beneficial disease control targets, such as the number of symptomatic patients receiving point-of-care testing or the costs of diagnosis of patients, would be important. Developing integrated targets may work as one of the mechanisms to incentivize collaboration and integration of services. A study from India illustrated how a vertical disease control programme with an explicit policy of strengthening local health systems helped to facilitate integration of vertical programmes.^35^

Technical guidelines for diagnostic and antimicrobial stewardship will need to be redesigned, despite possible differences of opinion between disease-specific technical experts.^36^^,^^37^ Currently, tuberculosis laboratories embedded within a well-structured vertical programme have clear policies and guidelines for testing, interpreting results and treating patients. If tuberculosis laboratory services are to be expanded, guidelines on the use of diagnostics, information reporting protocols and management structures need to be updated. Such integration will require acceptance of new roles and new ways of working by the staff in laboratory systems. It will also create opportunities for accessing a larger patient population with a wider spectrum of infections, along with engaging health-care providers from various specialties and government bodies from different sectors. This can only be achieved through coordinated planning by antimicrobial resistance and tuberculosis control programmes at the country level; for example, to include managing the expanded remit of staff and their training in the use of a wider set of technologies.

## Conclusions

Integration of the nascent antimicrobial resistance programmes within the well established vertical disease control programmes is currently limited. This results in missed opportunities for greater efficiency and better patient-centred care. The World Health Organization has highlighted gaps in coordination of information management systems in antimicrobial resistance programmes, such as for electronic reporting and tools for standardized surveillance.^38^

Given the shift from conventional diagnostic tools to newer point-of-care tests and the large investments in antimicrobial resistance surveillance,^14^ we need to review the current role of diagnostic laboratories associated with disease-specific programmes. As newer multiplex point-of-care tests become increasingly available, the concept of programmes limited to diagnosis of a single disease will require rethinking. We argue that the tuberculosis laboratory system, with its strong microbiology expertise and infrastructure, is particularly well placed to contribute towards antimicrobial resistance control. Nevertheless, integration of disease-specific programmes, which are not unique to tuberculosis, also faces longstanding barriers.

Many potential mutual benefits of integration exist, in terms of accelerated scale-up of diagnostic testing towards rational use of antimicrobial drugs as well as optimal use of resources. To scale-up activities, it would be prudent for governments to build on the existing regulatory frameworks, surveillance systems, infection control systems, laboratory infrastructure and human resources that are already in place to manage tuberculosis.^19^ Diagnostic tools, logistics and technologies for sharing data can be used to link programmes at the country level towards a stronger programme to control antimicrobial resistance including in tuberculosis. Not only would antimicrobial resistance programmes gain from the tuberculosis laboratory system, but tuberculosis programmes themselves would benefit from the political attention and funding currently being directed towards antimicrobial resistance.

In addition to the focus on budgets and resources, combined or integrated inter-programme activities bring other advantages. The main goal in partnerships in public health has been ensuring the future sustainability of programmes. By forging a partnership between antimicrobial resistance and tuberculosis control programmes within countries’ governing structures, common goals will be safeguarded through a strengthened oversight mechanism. Moreover, programme integration presents opportunities to direct the focus of policy-makers towards the issue of antimicrobial resistance, which has so far met with limited success.^39^ Advocacy efforts to influence pharmaceutical regulation, formulary restrictions and use of growth promoters in the veterinary industry could be intensified. Public health messages released by control programmes are useful catalysts for behavioural change in communities. Reinforcement of such messages from tuberculosis clinics, as well as hospitals and clinics involved in antimicrobial resistance control efforts, is likely to lead to faster and more durable changes in antibiotic use, attitudes to infection prevention and general health awareness.
